# Methanogens: Methane Producers of the Rumen and Mitigation Strategies

**DOI:** 10.1155/2010/945785

**Published:** 2010-12-30

**Authors:** Sarah E. Hook, André-Denis G. Wright, Brian W. McBride

**Affiliations:** ^1^Department of Animal & Poultry Science, University of Guelph, Guelph, ON, Canada N1G 2W1; ^2^Department of Animal Science, University of Vermont, Burlington, VT 05405, USA

## Abstract

Methanogens are the only known microorganisms capable of methane production, making them of interest when investigating methane abatement strategies. A number of experiments have been conducted to study the methanogen population in the rumen of cattle and sheep, as well as the relationship that methanogens have with other microorganisms. The rumen methanogen species differ depending on diet and geographical location of the host, as does methanogenesis, which can be reduced by modifying dietary composition, or by supplementation of monensin, lipids, organic acids, or plant compounds within the diet. Other methane abatement strategies that have been investigated are defaunation and vaccines. These mitigation methods target the methanogen population of the rumen directly or indirectly, resulting in varying degrees of efficacy. This paper describes the methanogens identified in the rumens of cattle and sheep, as well as a number of methane mitigation strategies that have been effective *in vivo*.

## 1. Introduction

Methane production through enteric fermentation is of concern worldwide for its contribution to the accumulation of greenhouse gases in the atmosphere, as well as its waste of fed energy for the animal. Methane is produced in the rumen and hindgut of animals by a group of *Archaea* known collectively as methanogens, which belong to the phylum *Euryarcheota*. Among livestock, methane production is greatest in ruminants, as methanogens are able to produce methane freely through the normal process of feed digestion. Much research has been directed toward methane abatement strategies to be used in ruminants and has been reviewed elsewhere [1–7]. Abatement strategies are often limited by the diet fed, the management conditions, physiological state and use of the animal, as well as government regulations; resulting in difficulties applying a one size fits all approach to the problem of enteric methane mitigation. To this end, the aim of this paper is to provide background on enteric fermentation and methanogens, as well as some of the methane abatement strategies that have shown efficacy *in vivo*.

## 2. Methane and Ruminants

Greenhouse gases such as carbon dioxide, methane, nitrous oxide, and ozone contribute to climate change and global warming through their absorption of infrared radiation in the atmosphere [8]. Methane is classified as a trace gas and is estimated to have a total global concentration of 1774 ± 1.8 parts per billion (ppb), with a total increase of 11 ppb since 1998 [9]. Methane is an especially potent trace gas due to its global warming potential, 25 times that of carbon dioxide, and its 12-year atmospheric lifetime; it is the second largest anthropogenic greenhouse gas, behind carbon dioxide [9, 10]. Also, methane is able to increase ozone in the tropospheric region of the atmosphere where the greenhouse effect occurs, and increase stratospheric water vapour, both of which can add to the radiative force of the gas by approximately 70% [8]. Globally, 50–60% of methane emissions are from the agricultural sector, specifically from livestock production operations; the principal source of methane is from ruminant animals [11, 12].

Domesticated ruminants, such as cattle, sheep, and goats produce as much as 86 million metric tonnes (Tg) of methane per year [13]. Approximately 18.9 Tg are from dairy cattle, 55.9 Tg are from beef cattle, and 9.5 Tg are from sheep and goats [13]. Data from Johnson and Ward [14], estimates the global yearly methane contribution of buffalo to be 6.2–8.1 Tg, 0.9–1.1 Tg from camels, and methane production within the hindgut of pigs and horses to be approximately 0.9–1.0 Tg and 1.7 Tg, respectively.

Methane is produced in the rumen as a product of normal fermentation of feedstuffs. Although methane production can also occur in the lower gastrointestinal tract, as in nonruminants, 89% of methane emitted from ruminants is produced in the rumen and exhaled through the mouth and nose [15]. As methane is exhaled into the atmosphere, the ruminant suffers a loss of ingested feed-derived energy of approximately 2–12%, depending upon the diet [14]. 

The loss of methane to the atmosphere varies based on the ruminant species. Estimates of diet-derived energy losses from methane for dairy cattle, range-cattle, and feedlot cattle vary from 5.5–9.0%, 6.0–7.5%, and 3.5–6.5%, respectively [14]. For buffalo and camels, a loss of diet energy in the form of methane ranges from 7.5–9.0% and 7.0–9.0%, respectively [14]. Estimates of methane losses from ruminants also vary based on geographical location, feed quality, feed intake, feed composition, and the processing of the feed [14]. The impact of dietary components on methane emissions will be discussed further in Section 4.1

## 3. Methanogens

### 3.1. Characteristics and Ecosystem

Methanogens belong to the domain *Archaea* and the phylum *Euryarchaeota *[16]. Unlike *Bacteria*, methanogens lack peptidoglycan in the cell wall, replaced by pseudomurein in *Methanobrevibacter *and *Methanobacterium*, heteropolysaccharide in *Methanosarcina,* and protein in *Methanomicrobium *[16]. All methanogens have coenzyme F_420_, which is a cofactor necessary for enzymes such as hydrogenase and formate dehydrogenase, and received its name due to its absorbance at 420 nm, which allows it to fluoresce blue-green at 470 nm [17]. Another coenzyme characteristic of methanogens is coenzyme M, which is either produced by the methanogens, such as *Methanobacterium*, or is required from an external source, which is the case for *Methanobrevibacter ruminantium* [18]. Coenzyme M, or 2-mercaptoethanesulfonic acid, is methylated to produce methane [19]. 

The cell characteristics can vary greatly among methanogens as well. *Methanobrevibacter ruminantium* is rod shaped with variable motility and is able to use hydrogen and carbon dioxide, and formate as substrates for methane production [16]. *Methanobacterium formicicum*, which is in the same order (*Methanobacteriales*) as *Methanobrevibacter*, can be rod or filament shaped without motility, and is able to use the same substrates as *Methanobrevibacter* [16]. *Methanomicrobium mobile* is rod shaped and is motile, using both hydrogen and carbon dioxide, as well as formate to produce methane [16]. Finally, *Methanosarcina barkeri* and *Methanosarcina mazeii* are both coccoid shaped, but without motility [16]. The order *Methanosarcinales* contains the only methanogens with cytochromes, and can grow on the broadest range of substrates [20]. Cytochromes are membrane-bound electron carriers that play a role in the oxidation of methyl groups to carbon dioxide [21]. *Methanosarcina barkeri* is able to produce methane from hydrogen and carbon dioxide, acetate, methylamines, and methanol, whereas *Methanosarcina mazeii* can use the same substrates except hydrogen and carbon dioxide [16]. 

Methanogens with cytochromes have a growth yield of 7 g per mole of methane on hydrogen and carbon dioxide, and have a doubling time of greater than 10 hours [20]. Methanogens without cytochromes have a growth yield of 3 g per mole of methane on hydrogen and carbon dioxide, and have a doubling time minimum of 1 hour [20]. More in-depth discussion of taxonomy can be found in Garcia et al. [22] and a review of methanogens with emphasis on cytochromes can be found in Thauer et al. [20].

Methanogens are not only confined to the rumen in cattle and other ruminants. There has been recent interest in the presence of methanogens in the intestine of humans and *Archaea* have been found using 454 pyrosequencing in higher abundance in the large intestine of obese individuals [23]. Real-time PCR has been used to detect *Methanobrevibacter smithii* and *Methanosphaera stadtmanae* from human feces [24], and methanogens *Methanobrevibacter gottschalkii*, *Methanobrevibacter thaueri*, *Methanobrevibacter woesei *and *Methanobrevibacter wolinii *have been cultured from the feces of horse, cow, goose, and sheep, respectively [25]. Finally, *Methanobrevibacter oralis* was isolated in subgingival sites of patients with periodontal disease [26].

### 3.2. Methane Production

The principal methanogens in the bovine rumen utilize hydrogen and carbon dioxide, but there is a group of methanogens of the genus *Methanosarcina* that grow slowly on hydrogen and carbon dioxide and therefore maintain a distinct niche by utilizing methanol and methylamines to produce methane [27, 28]. Formate, which is formed in the production of acetate, can also be used as a substrate for methanogenesis, although it is often converted quickly to hydrogen and carbon dioxide instead [27, 29]. Volatile fatty acids (VFA) are not commonly used as substrates for methanogenesis as their conversion into carbon dioxide and hydrogen is a lengthy process, which is inhibited by rumen turnover [19]. Therefore, methanogenesis often uses the hydrogen and carbon dioxide produced by carbohydrate fermentation, as VFAs are formed [27]. By removing hydrogen from the ruminal environment as a terminal step of carbohydrate fermentation, methanogens allow the microorganisms involved in fermentation to function optimally and support the complete oxidation of substrates [30]. The fermentation of carbohydrates results in the production of hydrogen and if this end product is not removed, it can inhibit metabolism of rumen microorganisms [30].

### 3.3. Strains of Importance

The methanogen population present in the rumen may differ depending on the ruminant species being examined. *Methanobrevibacter ruminantium *and *Methanomicrobium mobile *were found to be the major methanogens in the ovine rumen by Yanagita and coworkers [31] using 16S rRNA-targeted fluorescent *in situ* hybridization. Wright and colleagues [32] identified clones from ovine rumen fluid similar to cultivated methanogens from the order *Methanobacteriales*. In another study of ovine rumen methanogens, Wright et al. [33] identified clones from ovine rumen fluid similar to methanogens of the orders *Methanobacteriales* and *Methanomicrobiales*, as well as previously unidentified sequences. Methanogens from the rumen of sheep and cattle were examined in a study by Nicholson et al. [34] using temporal gradient gel electrophoresis and found to be similar to those of the order *Methanobacteriales* and *Methanosarcinales*, although previously uncultured methanogens were also detected. Wright and colleagues [35] also completed a diversity analysis of sheep from Venezuela and concluded that the majority of clones identified belonged to the genus *Methanobrevibacter*, with the largest group of clones being similar to *Methanobrevibacter gottschalkii*. 

In the bovine rumen, Whitford and coworkers [36] were able to detect *Methanobrevibacter ruminantium* as the largest group of methanogens in lactating dairy cattle fed total mixed ration, followed by *Methanosphaera stadtmanae*. Isolation of methanogens from grazing cattle by Jarvis et al. [37] suggested that *Methanomicrobium mobile* may be present at 10^6^ cells/ml. *Methanobacterium formicicum* was isolated as the second most common methanogen, followed by an isolate phenotypically similar to *Methanosarcina barkeri* [37]. *Methanobrevibacter *spp. was not identified in grazing cattle although it has been detected in cattle kept indoors and fed total mixed ration [36]. Wright and colleagues [38] identified methanogens from a clone library of the rumen fluid from feedlot cattle in Ontario, Canada fed a diet of predominantly corn. Clones were found to have greater than 95% sequence similarity with *Methanobrevibacter ruminantium*, *Methanobrevibacter thaueri, Methanobrevibacter smithii, *and *Methanosphaera stadtmanae* [38]. In the same study, a clone library was made from the rumen fluid of cattle from Prince Edward Island fed a diet of potato by-products [38]. Clones were found to have greater than 95% sequence similarity with *Methanobrevibacter smithii, Methanobrevibacter ruminantium, *and* Methanobrevibacter thaueri* [38]. Also, the rumen contents of cattle from Ontario and Prince Edward Island were found to have methanogen clones unique to the geographical location from which they originated, indicating that diet as well as geographical location of the host may play a role in the methanogen population diversity present.

Methanogen strain presence has also been investigated as it relates to feed efficiency. Recent work by Zhou and colleagues [39, 40] investigated the diversity of methanogens in the rumen of beef cattle with either high or low feed efficiencies. In the 2009 study, *Methanosphaera stadtmanae *and *Methanobrevibacter *sp. AbM4 were in greater numbers among the inefficient animals, while the total size of the rumen methanogen population was not significantly different between animals with different feed efficiencies [39]. In the 2010 study, where they also had cattle fed either a high- or low- energy diet, the high-energy diet was associated with the presence of *Methanobrevibacter smithii *SM9 and *Methanobrevibacter *sp. AbM4, while the predominant methanogen in the low-energy diet was *Methanobrevibacter ruminantium *[40]*. Methanobrevibacter smithii* was only found in high efficiency animals.

Furthermore, the location within the rumen from which the methanogens are detected plays a role in the methanogens identified. In a study by Shin and colleagues [41], a Korean cow was fed rice hull and concentrate, and samples from the rumen fluid, rumen solid, and rumen epithelium were removed. The predominant rumen methanogen in the rumen fluid and rumen epithelium was found to belong to the family *Methanomicrobiaceae* [41]. The rumen solid was predominantly made up of methanogens of the family *Methanobacteriaceae*, which is the methanogen family commonly detected within the bovine rumen [41].

### 3.4. Relationship with Other Microorganisms

Methanogens are known to have symbiotic relationships involving interspecies hydrogen transfer with rumen microorganisms, especially with rumen protozoa where the methanogens can be associated intracellularly and extracellularly [30]. Common protozoa in the bovine rumen found to have such a relationship are from the genera *Entodinium, Polyplastron*, *Epidinium*, and *Ophryoscolex*, while the methanogens most often associated with protozoa are from the orders *Methanobacteriales* and *Methanomicrobiales* [30]. Anaerobic fungi, such as *Neocallimastix frontalis,* have also been found to have a relationship with methanogens involving interspecies hydrogen transfer whereby the fungi's enzymatic activity has increased and metabolism has shifted towards acetate production [42–44].

## 4. Methane Reduction Strategies

The topic of the relationship that methanogens have with other microorganisms in the rumen is especially important when considering methane mitigation strategies. Methane mitigation is effective in one of two ways: either a direct effect on the methanogens, or an indirect effect caused by the impact of the strategy on substrate availability for methanogenesis, usually through an effect on the other microbes of the rumen. Both approaches will be discussed here with focus on strategies that have shown efficacy *in vivo* (see Table 1).

### 4.1. Dietary Composition

The components of the diet fed, especially type of carbohydrate, are important for methane production as they are able to influence the ruminal pH and subsequently alter the microbiota present [45]. Ellis et al. [11] were able to predict methane production in dairy and beef cattle based on dry matter intake, neutral detergent fibre, and lignin content of the diet, measurements easily acquired from farms, with an *R* ^2^ of 0.71. The digestibility of cellulose and hemicellulose are strongly related to methane production, more so then soluble carbohydrate [46]. In a study by Holter and Young [46], a positive relationship was found between digestibility of hemicellulose and methane output in forage fed nonlactating cows. A negative relationship was found between digestibility of cellulose and methane output [46]. Sauvant and Giger-Reverdin [47] found the relationship between methane production and proportion of concentrate in the diet to be curvilinear, with methane losses of 6-7% of gross energy (GE) being constant at 30–40% concentrate levels in the diet and then decreasing to 2-3% of GE with a concentrate proportion of 80–90%. The starch component of the diet is also known to promote propionate formation, through a shift to amylolytic bacteria, and a reduction in ruminal pH, leading to a decrease in methanogenesis [48]. Johnson and Johnson [45] stated that the digestion of cell wall fiber increases methane production, by increasing the amount of acetate produced in relation to propionate. The increase in methane output is due to the fermentation of acetate, which provides a methyl group for methanogenesis [49]. Grinding forage feed before it is ingested by the cows also seems to decrease the production of methane, presumably by increasing the rate of digestion and flow through the gastrointestinal tract, thus limiting the time available for methane to be produced within the rumen [45]. 

It is important to note that increasing the amount of rapidly fermentable carbohydrates in a diet can increase the rate of passage from the rumen, as well as lower the ruminal pH (see Table 1). Increased passage rates can shift methanogenesis to the hind gut, as well as to the manure, possibly off setting any reductions in ruminal methane outputs [50]. Further, the ruminal digestion of rapidly fermentable carbohydrates can increase the production of VFAs. If VFA production is greater than absorption, the pH in the rumen will drop, leading to subacute ruminal acidosis (SARA) and disruption of the rumen microbiota [51]. 

### 4.2. Lipids

Lipids, such as fatty acids and oils, are options for feed supplementation that have been investigated both *in vitro* and *in vivo* for their effects on methanogenesis. Increased lipid content in the feed is thought to decrease methanogenesis through inhibition of protozoa, increased production of propionic acid, and by “biohydrogenation of unsaturated fatty acids” [45]. Unsaturated fatty acids may be used as hydrogen acceptors as an alternative to the reduction of carbon dioxide [45]. Also, fatty acids are thought to inhibit methanogens directly through binding to the cell membrane and interrupting membrane transport [52]. Interestingly, Kong et al. [53] detected *Archaea* using fluorescence *in situ* hybridization (FISH) in the rumen of dairy cows supplemented with flaxseed, and did not find any obvious differences in the proportion of *Archaea* present with flaxseed addition. The authors stated that it was possible fatty acid supplementation was affecting activity instead of quantity of methanogens.

A meta-analysis of methane output with lipid supplementation in lactating dairy cows found a 2.2% decrease in methane per 1% of supplemented lipid in the diet [54]. In cattle and sheep, Beauchemin et al. [55] found an association of 5.6% methane reduction per percentage unit of lipid added to the diet. There are many factors that may account for varying effects of lipids on methane abatement, such as the ruminant species, experimental diet, and the type of lipid used. An excellent review of *in vivo* experiments using lipid supplementation to investigate methane abatement can be found in Martin et al. [6]. 

#### 4.2.1. Fatty Acids

A number of fatty acids have been investigated *in vivo* for methane suppressing effect. Myristic acid was found to reduce methane by 22% in sheep fed a forage-based diet and 58% in a concentrate-based diet when 50 mg/kg DM was used [56]. Odongo et al. [57] measured a 36% methane reduction in dairy cattle fed a total mixed ration with 5% myristic acid supplementation on a dry matter (DM) basis. In *vitro* studies have found fatty acids used in combination have the greatest suppression of methanogenesis due to a synergistic effect [52, 58]. Therefore, it is likely that oil supplementation would provide a more dramatic depression of methane production than individual fatty acids [58]. 

#### 4.2.2. Oils

Oils extracted from plant sources usually contain a favourable amount of medium- to long-chain fatty acids [58, 59]. Refined soy oil fed to beef bulls at 6% inclusion reduced methane production by 39% in terms of litres per day (l/d) [60]. Sunflower oil is more often studied and has resulted in an 11.5–22.0% reduction in methanogenesis [59, 61]. Sunflower oil has also been combined with linseed oil at a ratio of 1 : 3 and fed to sheep on a pasturebased diet in a dose-response trial, but at 1.2–5% oil inclusion on a dry matter basis, there was no significant reduction in methanogenesis [62]. Linseed oil supplemented at a level of 5% of DM to lactating dairy cows resulted in a 55.8% reduction in grams of methane per day [63]. Coconut oil is the most popular oil for methane abatement experiments and has been found to induce significant reductions in methanogenesis, although the extent of the reduction varies from 13–73%, depending on the inclusion level, diet, and ruminant species used [60, 64, 65]. Since coconut oil has a ratio of lauric to myristic acid of 2.6 : 1.0, similar to the effective ratios for methane abatement of 4 : 1, 3 : 2, and 2.5 : 2.5 found *in vitro* by Soliva et al. [58], it is expected that this oil would provide significant reductions in methanogenesis *in vivo*. Palm kernel oil has a ratio of lauric to myristic acid of 3 : 1, suggesting a greater efficacy for methane abatement compared to coconut oil, but to our knowledge, there are currently no published reports of palm kernel oil supplementation *in vivo.* In an *in vitro* study by Dohme et al. [66], coconut oil reduced methane by 21% while palm kernel oil reduced methane by 34%, providing more evidence that palm kernel oil may be more efficacious. However, it is important to note, that *in vivo* studies involving oil supplementation are often accompanied by a reduction in dry matter intake, which can also result in reduced methane production [65].

#### 4.2.3. Other Lipid Sources

Other lipid sources, such as tallow and seeds, have also been investigated for methane suppressing effects. Beauchemin et al. [61] supplemented heifers with 34 g of tallow per kg DM and found an 11% reduction in g of methane per kg DMI. Jordan et al. [67] supplemented beef bulls with whole soybean at an inclusion level of 27% DM and, despite palatability issues resulting in up to 60% refusal, found a 25% reduction of liters methane per day. Beauchemin et al. [61, 68] conducted two experiments using sunflower seed supplementation with heifers and dairy cows and found a 23% and 10.4% reduction in methanogenesis, respectively. Beauchemin et al. [68] also supplemented dairy cows with flaxseed and canola seed at 3.3% (DM basis) and reductions in methane were found to be 17.8% and 16.0%, respectively, as g/kg DMI [68]. Machmüller et al. [65] found reductions in methane on a kg live weight basis from supplementation of rapeseed, sunflower seed, and linseed of 19%, 27%, and 10%, respectively, in growing lambs. Finally, Grainger et al. [69] fed 2.61 kg (DM basis) of whole cottonseeds to lactating cows and found the average reduction in methane over the twelve-week experiment was 2.9% per 1% fat addition, with 1.5% reduction at week three and 4.4% at week twelve. 

 No matter what the lipid form used for supplementation, it is important to consider the ruminant species and the diet being examined, as methane reductions can vary depending on the feed components present (see Table 1) [6]. Further, lipid inclusion can affect palatability, intake, animal performance, and milk components, all of which can have implications for practical on-farm use [57, 67]. Finally, the majority of *in vivo* experiments conducted to investigate lipids as methane abatement strategies are short-term, making it nearly impossible to draw conclusions about long-term repressive effects. Therefore, long-term supplementation experiments need to be conducted to thoroughly gauge the efficacy of lipid supplementation as an abatement strategy.

### 4.3. Defaunation Treatment

Defaunation, which is the removal of protozoa from the rumen, has been used to investigate the role of protozoa in rumen function, and also to study the effect on methane production. Rumen protozoa, as stated previously, share a symbiotic relationship with methanogens, participating in interspecies hydrogen transfer, which provides methanogens with the hydrogen they require to reduce carbon dioxide to methane [70]. It has been estimated that the methanogens associated with the ciliate protozoa, both intracellularly and extracellularly, are responsible for 9 to 37% of the methane production in the rumen [70–72]. For this reason, treatments that decrease the protozoal population of the rumen, may also decrease the protozoa-associated methanogen population and therefore, decrease the methane production within the rumen. Treatments that have been used include copper sulphate, acids, surface-active chemicals, triazine, lipids, tannins, ionophores, and saponins [19]. It has been suggested that the effect of defaunation on methane output is diet dependent. Hegarty [73] found that defaunation reduced methane output 13%, but the magnitude of reduction varied with diet. The greatest reduction in methane production with defaunation was measured on a high-concentrate diet, likely because protozoa are the predominant source of hydrogen for methanogenesis on starch-based diets. Although, Hegarty et al. [74] also found that there was no main effect of protozoa on rumen methane production, when investigated in chemically-defaunated, defaunated from birth, and faunated lambs. Another consideration is whether there are long-term effects of defaunation on methanogenesis (see Table 1). Morgavi et al. [75] found methane reductions due to defaunation to last more than two years, but a study of ionophore supplementation by Guan et al. [76] found that reductions in rumen methanogenesis were short-lived and hypothesized this was due to adaptation of ciliate protozoa. Finally, maintenance of defaunated animals can be difficult. A recent study found that transfer of viable protozoa to defaunated animals does not occur readily through contact with feed or feces of faunated animals, nor with direct contact with faunated animals, but does occur through contaminated water [77]. 

### 4.4. Vaccine

Another methane reduction strategy that is being investigated is the development of a vaccine that would stimulate the ruminant's immune system to produce antibodies against methane-producing methanogens [78]. In a study by Wright and colleagues [78], two vaccines were developed, named VF3 (based on three methanogen strains) and VF7 (based on seven methanogen strains), which produced a 7.7% methane reduction per dry matter intake, despite only approximately 20% of the methanogen population being targeted. The same research group also created a vaccine based on five methanogen strains that was administered in three vaccinations to sheep [79]. Although the vaccine targeted 52% of the methanogens present in the rumen of the sheep, methane output went up 18% with vaccination, leading the authors to believe that the vaccine was not targeting the methanogens capable of producing most of the methane. Another consideration when using vaccines against methanogens is that the rumen methanogen population present can differ based on diet and geographical location of the host, making a single-targeted approach difficult [38]. 

An additional vaccine has recently been developed using subcellular fractions of *Methanobrevibacter ruminantium *M1 [80]. Twenty sheep were vaccinated and then revaccinated three weeks later and the antisera was found to cause agglutination of methanogens and decrease growth and methane production *in vitro*. *In vivo* testing of the efficacy of the vaccine on methanogens was not conducted (see Table 1).

### 4.5. Monensin

Monensin, an antibiotic produced by *Streptomyces cinnamonensis*, is marketed in North America to increase feed efficiency and weight gain, increase milk production, and decrease milk fat [81]. More recently, interest has been renewed in monensin as a mitigation strategy for methane production, as it is known to inhibit gram-positive microorganisms responsible for supplying methanogens with substrate for methanogenesis. The effects caused by monensin on the microbial cell are mediated by its ability to interfere with ion flux [82, 83]. Monensin selects for gram-negative microorganisms, which causes a shift towards propionate production in the rumen [82, 83]. For this reason, it is hypothesized that monensin does not affect methane production by inhibiting methanogens, but instead inhibits the growth of the bacteria, and protozoa, providing a substrate for methanogenesis [82–85]. This statement is strengthened by the fact that when rumen fluid was dosed with monensin *in vitro*, methane production decreased until a supply of hydrogen was given, at which time methane production resumed [83]. 

The reductions in methanogenesis following ionophore supplementation vary from minor to 25%, with differing outcomes for the duration of these effects [45]. In a study designed to measure methane output in lactating dairy cows receiving monensin supplementation, cows were fed monensin-supplemented feed for 3 weeks after a transitional period, fed a monensin-free diet for 5 months, and then fed monensin-supplemented feed for another 3 weeks [81]. It was found that although during the first treatment with monensin the cows had decreased feed intake, increased propionate production, and decreased methane outputs, the second treatment of monensin did not cause the previously seen effects. There was confounding within this study of treatment and animal, but the authors stated that adaptation of the rumen microflora to monensin may have occurred during the first treatment, inhibiting the effect of the drug during the second treatment. 

Guan et al. [76] investigated the use of monensin in steers and the effect of supplementation on methane output. Steers were fed either a low-concentrate diet or a high-concentrate diet while supplemented with monensin. For the low-concentrate diet, an initial reduction in methane output, as measured with sulfur hexafluoride (SF_6_) tracer gas, was found of 27% over the initial four weeks, in combination with a reduction in the ciliate protozoal population of 77% [76]. For the high-concentrate diet, within the first two weeks there was a 30% reduction in methane output along with an 83% reduction in the ciliate protozoal population [76]. The methane levels returned to baseline and the protozoal numbers returned to baseline as well after six and four weeks, respectively. The authors concluded that the affect of monensin on methane levels in the rumen is related to the ciliate protozoal population and as this population adapted to monensin, the methane levels in the rumen returned to pretreatment levels.

More recently, long-term monensin supplementation was investigated in lactating dairy cows fed a milking cow total mixed ration [86]. Twenty-four cows were pair-fed and baseline measurements of methane output were measured. Monensin supplementation was included in the diet of half of the paired animals while the other half was fed the same diet without monensin, and methane output was measured for each pair monthly for six months. Monensin treatment was found to cause a 7–9% reduction in methane output versus control cows and this reduction was sustained for the entire treatment period with no adaptation detected [86]. In conjunction with this experiment, rumen samples were obtained for molecular analysis of changes in the methanogen population with monensin supplementation [87]. No significant differences in the number or diversity of methanogens were found, confirming that monensin is able to suppress methanogenesis through an indirect effect on methanogens.

Therefore, although monensin supplementation has been shown to effectively reduce methane output in ruminants, there are a few factors that may impact the efficacy (see Table 1). First, there appears to be differences in the degree of abatement depending on the diet and animal used [76, 86]. Also, the ciliate population present in the rumen may affect the outcome of supplementation, with adaptation being a possibility [76]. Finally, monensin has been banned in the European Union, so an alternate methane abatement method would be required in those countries.

### 4.6. Plant Compounds

The three main plant compounds effective at reducing methane emissions *in vitro* are condensed tannins, saponins, and essential oils. *In vivo*, the efficacy of these compounds varies in terms of methane abatement. 

Condensed tannins are thought to directly inhibit methanogens, as well as indirectly limit methanogenesis through a reduction in hydrogen availability [88]. Condensed tannin-containing *Lespedeza cuneata* was fed to goats *ad libitum* and found to reduce methane 57% in terms of g/kg DMI, compared to goats fed a mixture of *Digitaria ischaemum *and *Festuca arundinacea* [89]. Sheep consuming 41 g of tannin-containing *Acacia mearnsii *per kg DM were found to have a 13% reduction in methanogensisis [90]. Tannin-containing *Callinada calothyrsus *and *Fleminga macrophylla* also reduced methane 24% in lambs [91], but an extract of condensed tannin from *Schinopsis quebrachocolorado* [92] and tannin-containing sorghum silage [93] fed to cattle did not suppress methanogenesis. 

Saponins have been shown *in vitro* to inhibit protozoa, as well as limit hydrogen availability for methanogensis [94]. A recent study by Holtshausen et al. [95] supplemented cows with whole-plant *Yucca schidigera *powder at 10 g/kg DM or whole-plant *Quillaja saponaria* powder at 10 g/kg DM, both of which contain saponin. The authors stated that previous studies *in vitro* had found reductions in methane at higher inclusion levels (15 g/kg DM and greater), but these high levels were avoided *in vivo* in order to minimize effects on digestibility [94]. No effect of the plant supplementation was found *in vivo* and the authors concluded that the *in vitro* reductions in methane were likely due to reduced feed digestion and fermentation [95]. This makes *in vivo* supplementation difficult because higher feeding levels may be required to measure reductions in methane output, but these reductions would be at a cost to feed-digestibility.

Essential oils have antimicrobial activities that act in a similar way to monensin by inhibiting gram-positive bacteria [96, 97]. In this way, essential oils can reduce the amount of available hydrogen for methanogensis. Few *in vivo* studies have been conducted, but one study by Beauchemin and McGinn [98] where heifers were fed 1 g/d of essential oil and spice extract found no effect on methane output and a negative effect on feed digestibility. 

Clearly, more research in necessary *in vivo* with essential oils, as well as condensed tannins and saponins, to determine the optimal dosage where methanogenesis is reduced without side effects on digestibility (see Table 1). Also, long-term studies are required to determine whether the microbes are able to adapt to supplementation and resume methanogenesis at baseline levels. Finally, it is important to study whether any residues of supplementation appear in milk or meat to make this a viable option for methane abatement in production animals [97]. 

### 4.7. Organic Acids

*In vivo* effects of organic acid supplementation on methane abatement are variable. Wood and colleagues [99] supplemented 100 g/kg fumaric acid in the free or encapsulated form to growing lambs and found a 62% and 76% reduction in methane output, respectively. Fumaric acid was also fed to growing beef cattle at 175 g/d, steers at 80 g/d, and wethers at 4–10 g/100 g (DM basis), but was not found to significantly reduce methane emissions, although suppression of DMI was found at higher inclusion levels [59, 100]. Beef heifers were supplemented with 3.75% and 7.5% malic acid on a DM basis and methane output reductions of 3% and 9% as g/kg DMI were measured, respectively [101]. The authors stated that the effect of organic acid supplementation on methane abatement appears to be influenced by diet, with greater abatement when high-concentrate diets are fed. This is due to a greater effect on the acetate-to-propionate ratio in the rumen, in addition to its ability to act as a hydrogen sink [101]. Based on the *in vivo* studies presented here, it appears that organic acids may provide beneficial effects in terms of methane abatement, but further *in vivo* experiments need to be conducted to determine the optimal conditions for use (see Table 1). Additionally, long-term supplementation studies need to be conducted to confirm that any benefits observed are lasting.

## 5. Summary

For more than two decades, researchers have been working to identify, quantify, and inhibit methanogens and methanogenesis through various methane mitigation strategies. Although a great deal of information has been gleaned from these experiments, including identification of a number of methanogen strains in the rumens of cattle and sheep around the world, as well as mitigation strategies with varying degrees of feasibility and efficacy, there is still more research to be done in this field. Also, many methane mitigation strategies work through an indirect effect on methanogens, by limiting substrate availability for methanogenesis. By targeting the methanogens directly, there may be a greater reduction in methanogenesis, as well as a more sustainable reduction, making strategies such as the use of vaccines and dietary fatty acids, which inhibit methanogens and protozoa, especially promising.

## Figures and Tables

**Table 1:** Methane abatement strategies, mechanism of abatement, and considerations for use.

Methane abatement strategy	Mechanism of abatement activity	Considerations when selecting abatement strategy
Dietary composition		
Increase hemicellulose/starch Decrease cell wall components Grinding	Increased passage rate; greater proportion propionate versus acetate; reduced ruminal pH	Shift methanogensis to hind gut or manure, risk of subacute ruminal acidosis (SARA)
		
Lipids		
Fatty acids Oils Seeds Tallow	Inhibition of methanogens and protozoa; greater proportion propionate versus acetate; biohydrogenation	Effect on palatability, intake, performance, and milk components; varies with diet and ruminant species; long-term studies needed
		
Defaunation		
Chemical Feed additives	Removes associated methanogens; less hydrogen for methanogenesis	Adaptation of microbiota may occur; varies with diet; maintenance of defaunated animals
		
Methanogen Vaccine	Host immune response to methanogens	Vaccine targets; diet and host geographical location differences
		
Monensin	Inhibits protozoa and gram-positive bacteria; lack of substrate for methanogenesis	Adaptation of microbiota may occur; varies with diet and animal; banned in the EU
		
Plant Compounds		
Condensed tannins Saponins Essential oils	Antimicrobial activity; reduced hydrogen availability	Optimum dosage unknown; more *in vivo* research needed; long-term studies needed; may affect digestibility; residues unknown
		
Organic Acids		
Fumarate Malate	Hydrogen sink, greater proportion propionate versus acetate	Varies with diet; more *in vivo* research needed; long-term studies needed; may affect digestibility
